# In Vivo Role of Two-Component Regulatory Systems in Models of Urinary Tract Infections

**DOI:** 10.3390/pathogens12010119

**Published:** 2023-01-10

**Authors:** Giuseppe Valerio De Gaetano, Germana Lentini, Agata Famà, Francesco Coppolino, Concetta Beninati

**Affiliations:** 1 Department of Human Pathology, University of Messina, 98124 Messina, Italy; 2 Department of Biomedical, Dental and Imaging Sciences, University of Messina, 98124 Messina, Italy; 3 Scylla Biotech Srl, 98124 Messina, Italy

**Keywords:** UTI, TCSs, mouse models, mutants, virulence genes

## Abstract

Two-component signaling systems (TCSs) are finely regulated mechanisms by which bacteria adapt to environmental conditions by modifying the expression of target genes. In bacterial pathogenesis, TCSs play important roles in modulating adhesion to mucosal surfaces, resistance to antibiotics, and metabolic adaptation. In the context of urinary tract infections (UTI), one of the most common types infections causing significant health problems worldwide, uropathogens use TCSs for adaptation, survival, and establishment of pathogenicity. For example, uropathogens can exploit TCSs to survive inside bladder epithelial cells, sense osmolar variations in urine, promote their ascension along the urinary tract or even produce lytic enzymes resulting in exfoliation of the urothelium. Despite the usefulness of studying the function of TCSs in in vitro experimental models, it is of primary necessity to study bacterial gene regulation also in the context of host niches, each displaying its own biological, chemical, and physical features. In light of this, the aim of this review is to provide a concise description of several bacterial TCSs, whose activity has been described in mouse models of UTI.

## 1. Introduction

Urinary tract infections (UTI) are one of the most common bacterial infections affecting millions of people in the world each year [1]. UTI are a significant cause of morbidity and is mainly diagnosed in women during their lifetime [2,3]. The higher prevalence of this infection in women is thought to be due to anatomical differences between the male and female genitourinary tracts. Women have a significantly shorter urethra than men and, for this reason, bacteria must travel a shorter distance to reach the bladder from the perianal area, which is the primary colonization site of many uropathogens [4]. The risk for UTI increases with other variables, including age, sexual activity, concomitant pathologies, and previous episodes of UTI [3]. Annually, UTI account for a huge number of visits to physicians and emergency departments as well as hospital admissions and are becoming difficult to treat owing to increased antibiotic resistance [5,6,7,8]. Clinically, UTI can be classified as complicated or uncomplicated. Complicated UTI are associated with predisposing conditions such as urinary tract obstruction, urine retention due to functional abnormalities, and catheterization [9]. These factors may lead to increased bacterial growth and damage of mucosal layers [10]. In this scenario, pathogens may ascend through the ureters into the kidneys, causing renal dysfunction and often leading to pyelonephritis [11]. If patients are not timely treated, bacteremia and septicemia may ensue [12]. On the other hand, uncomplicated UTI affect people without urinary tract abnormalities and tend to involve only the bladder and the lower urinary tract without invasion of the upper urinary tract or systemic infection [13]. 

The most common agents of complicated and uncomplicated UTI are uropathogenic *Escherichia coli* (UPEC), *Enterococcus faecalis*, *Klebsiella pneumoniae*, *Staphylococcus aureus*, *Proteus mirabilis*, group B Streptococcus (GBS), and *Pseudomonas aeruginosa* [14,15,16,17]. Frequent treatment of patients with antibiotics can result in changes in the normal microbiota of the vagina and the gastrointestinal tract leading to increased risk of urinary tract colonization by multidrug-resistant uropathogens [18,19,20]. For example, among *Enterobacteriaceae*, *E. coli* and *K. pneumoniae* have both acquired resistance to β-lactam antibiotics, while *Enterococcus* spp. have developed resistance to vancomycin [21]. 

The initial pathogenetic event in UTI is bacterial adhesion by means of pili and other surface structures and colonization of the urethra [22]. Subsequently, uropathogens must migrate to the bladder and, in this process, they can take advantage of factors that favor their motility, such as increased expression of flagella [23,24,25]. Production of toxins [26,27,28] and proteolytic enzymes [29,30] promotes access of uropathogens to host nutrients. Local replication and avoidance of immune surveillance [31,32,33,34] is also required to allow uropathogens to cause persistent infection, ascend to the kidneys, and access the bloodstream. A common strategy is the pathogen ability to form biofilms either on indwelling devices or directly on the surface of urothelium [35,36]. This mechanism protects uropathogens from the effect of host immunity and antibiotics favoring their persistence in the urinary tract [37,38,39]. Another strategy is the use of various virulence factors to bind to host ligands present in tissues or on the surface of urinary catheters, such as fibrinogen [40], fibronectin [38,41], laminin [42], plasminogen [42,43], and collagen [44]. UPEC and *K. pneumoniae*, for example, bind to uroplakins which are expressed on the apical membrane of umbrella cells [45]. These data show that urothelial receptors are important players in uropathogen colonization and/or invasion and can be considered as potential targets to reduce recurrent urinary tract infections (rUTI), as defined by three or more UTI in the course of one year [46]. In subverting host defenses, several uropathogens, such as UPEC, invade the urothelial cells and form intracellular bacterial communities (IBCs) by multiplying in the cytoplasm before pouring into the lumen of the bladder [46,47]. In other circumstances, uropathogens may invade deeper layers of the urothelium remaining inside intracellular niches in an inactive metabolic state to be re-activated after exfoliation of the highest layers of urothelium. This response is accelerated by neutrophil recruitment to the urothelium and host secretion of inflammatory cytokines [48,49,50]. Uropathogens may also change their shapes to circumvent the host immune system. Filamentation is a process that blocks septation ring formation and division in UPEC when they emerge from intracellular bacterial communities. In this manner, UPEC resist to neutrophil phagocytosis and can invade other urothelial cells [50]. Alternatively, the interactions between flagella and the host urothelial surface induce morphological changes in the outer membrane of *P. mirabilis* resulting in the acquisition of a hyper-flagellated phenotype required for increasing bacterial motility and propagation of infection [51,52].

## 2. Two-component Systems

**Figure 1:**
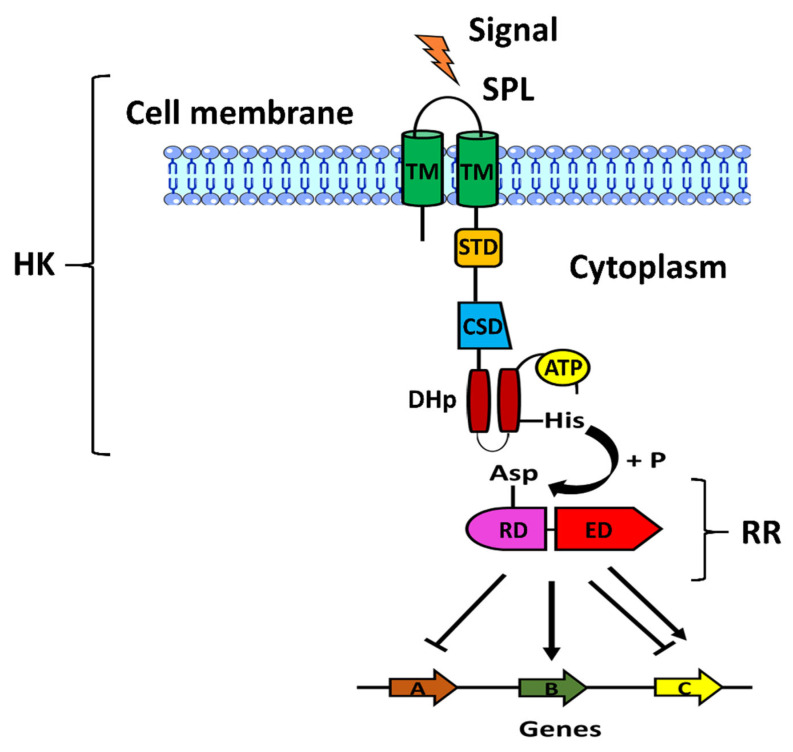
Representation of the main components of a two-component system (TCS). The sensor Histidine Kinase (HK) senses a signal (orange thunderbolt) via a Signal Peptidic Linker (SPL) which is associated with two Transmembrane (TM) helices. The sensing domain is joined to the Signal Transduction Domain (STD), the Cytoplasmic Sensor Domain (CSD), the ATP catalytic domain (ATP), and the Dimerization Histidine phosphotransfer domain (DHp). The conserved histidine residue (His) is first auto-phosphorylated and subsequently transferred to the conserved aspartate residue (Asp) of the Receiver Domain (RD) of the cognate Response Regulator (RR). Phosphorylation of the RR induces the Effector Domain (ED) to bind to its target genes and regulate their expression by inhibiting (A), inducing (B) or both (C) based on the nature of the inducing stimulus.

In bacterial pathogenesis, a central role is played by two-component systems (TCSs). They are involved in sensing various types of stimuli present in the environment such as osmotic pressure, membrane stress, the presence of specific host molecules, pH, antibiotics, and cues associated with intracellular location. TCSs are composed of a sensor histidine kinase (HK) and a response regulator (RR) [53]. The canonical structure of a HK is composed of four main domains: a sensor domain, comprising two transmembrane (TM) helices, an intracellular signal transduction domain (STD) also defined as HAMP (commonly found in Histidine kinase, Adenyl cyclase, Methyl-accepting proteins, and Phosphatase), a cytoplasmic sensor domain, and a conserved intracellular kinase domain that mediates both autophosphorylation and the transfer of a phosphoryl-group to the RR (Figure 1) [54]. The aforementioned kinase domain is composed of the Dimerization and Histidine phosphorylation subdomain, named as DHp, and the Cataytic and ATP-binding (CA) subdomain [54]. The sensor domains of HK are cytosolic or located on the cell surface [54]. The majority of sensor domains can have three types of structural folding: mixed αβ, all-helical or β-sandwich [54]. The response regulators have a simpler structure than HKs. A prototypical RR is made of two domains: a receiver domain and an effector domain. While the first one accepts a phosphoryl group transferred by a cognate HK, the second one triggers the signaling events. Unlike the effector domains, the receiver domains have a sequence where an aspartate residue is well conserved and is phosphorylated by HK. This event results in conformational changes in the receiver domain with the consequent activation of the effector domain. Structurally, the receiver domain has a conserved (βα)_5_ fold, in which a five-stranded β-sheet is surrounded by α-helices [55]. After the activation of RRs by phosphorylation, several structural changes are induced in some of the α and β elements positioned in the receiver domains, particularly in correspondence of the conserved Thr/Ser and Tyr/Phe key residues [54,56]. Effector domains, also known as output domains, have generally DNA-binding properties and are classified into several subfamilies based on predicted domain structures. The most common effector domains have a winged Helix-Turn-Helix (wHTH) motif or a Helix-Turn-Helix (HTH) motif [57,58,59]. In general, bacteria require numerous TCSs, depending on the specific inputs detected by the various HK. Indeed, metabolic adaptations to the host milieu are dependent on the integration of various TCSs-dependent molecular pathways that need to be investigated in vitro and in vivo.

In addition to information obtained by proteomics and metabolomics analysis, gene expression studies are crucial to understand how TCSs act on the genome. The typical approach is to verify gene expression in mutants deleted either in the RR, HK or both, in comparison with the wild-type (WT) parental strain [60,61]. Furthermore, mutant strains overexpressing the RR may be greatly suitable to examine target gene expression when the activating stimulus is unknown [62]. Various tools are also available to map TCS-targeted sequences and to define signaling networks across different bacterial species or types of infection [63]. In this manner, researchers can understand whether RRs may function as activators, repressors or both. Although biopsies are not frequently performed in patients with UTI, transcriptomic analyses of bacteria present in urine samples from patients provided useful information to analyze in vivo gene expression [64]. Moreover, various mouse models have been developed to study the pathogenesis of UTI [65]. Therefore, identification of TCS-activating stimuli, bacterial signaling mechanisms, and associated transcriptional changes seems relevant to the development of approaches to control infections [66]. In light of this, the aim of this review is to provide a schematic description of the function of some TCSs used by uropathogens focusing on those investigated in mouse models of UTI (Table 1).

### 2.1. Uropathogenic Escherichia coli

Uropathogenic *Escherichia coli* (UPEC) is the major cause of UTI in humans with millions of hospitalizations and emergency visits each year [67,68]. UPEC uses several virulence factors to survive in the urinary tract such as pili, hemolysins, and flagella [23,26,69]. The best characterized *E. coli* virulence factors involved in UTI pathogenesis are surface-exposed or secreted [70] and are schematically listed in Table 2.

UPEC belong to the extra-intestinal pathogenic strains (ExPEC) group, which is characterized by a high degree of genetic heterogeneity with an extensive range of different virulence factors. Almost all UPEC strains express type 1 pilus-associated protein FimH that binds to a variety of extracellular matrix components as well as mannose-containing host glycoproteins on umbrella cells [67,71,72]. Moreover, several adhesins, such as F1C pili, S pili, and the autotransporter proteins Upa, mediate the development of biofilms [73,74,75,76]. Biofilm assembly on urinary catheters renders UPEC extremely resistant to antibiotics and host immune responses [76]. Among host receptors implicated in mediating invasion of host cells, integrins [77], the complement regulatory protein CD46 [78], the complement decay-accelerating factor CD55 [79], and the flagellin sensor Toll-like receptor 5 (TLR-5) play major roles [80].

Furthermore, UPEC enter into bladder cells by dysregulating the actin cytoskeleton and promoting a zipper-dependent endocytosis pathway [81]. Once inside bladder cells, UPEC can meet alternative fates, including going back into the extracellular environment [82], replicate intracellularly to create IBCs [83,84] or persist intracellularly without multiplying [85,86]. Intracellular persistence is an effective strategy to avoid the action of antibiotics because of the intrinsic impermeability of the urothelial barrier and the inability of most drugs to cross cell membranes [87]. It is interesting to note that, in order to adapt to host microenvironments, InPEC express virulence factors similar to those expressed by UPEC, including fimbrial adhesins [88,89]. Several of these adhesins are also expressed by other uropathogens, such *P. mirabilis* and *K. pneumoniae*, and are considered as suitable targets for anti-adhesive therapeutics [90,91].

While several TCSs have been described in *E. coli*, relatively few have been analyzed in the context of UTI (Table 3).

Since osmolarity can vary considerably in the urinary tract, UPEC has developed strategies to sense external osmolar fluctuations and adapt to these changes [92,93]. One way to accomplish this is the EnvZ-OmpR TCS, which senses osmotic changes, upregulates the expression of the outer membrane porin proteins OmpF or OmpC, and modulates the expression of type 1 pili via activation of the transcriptional regulator OmpR [94,95,96]. The clinical isolate NU149, deleted of the *ompR* gene (Δ*ompR*), drastically reduced its rate of growth, compared to the WT parental strain, in the presence of increasing NaCl concentrations, whereas complementation with a functional *ompR* gene restored bacterial growth at WT levels [97]. Furthermore, growth of Δ*ompR* bacteria was further impaired when exposed to high NaCl concentrations in the presence of an acidic pH, thus mimicking the environmental conditions found in human urine. High concentrations of urea and sucrose in the growth medium also led to reduced growth of the Δ*ompR* strain. The phenotype of Δ*ompR E. coli*, was mimicked by strains carrying a single point mutation at position 55, the site of EnvZ-mediated phosphorylation [97].

The mouse urine has a pH ranging from pH 5.5 to 7.0 and osmolarities ranging from 350 to 600 mM [97]. In in vivo studies performed after inoculation of mice with *ompR* mutants, viable Δ*ompR* bacteria recovered from the bladder were markedly reduced at one-day post-challenge compared with WT bacteria, while there was only a tendency towards lower Δ*ompR* numbers in the kidneys. On the contrary, at the fifth day post-infection, viable Δ*ompR* bacteria were more drastically reduced in the kidneys than in the bladder compared with WT bacteria. Again, complementation of Δ*ompR* bacteria successfully restored the viable counts to WT levels [97]. These studies demonstrate the relevant role played by the response regulator OmpR in promoting UPEC ascension along the urinary tract. Furthermore, these data are in agreement with those of other papers, where mutations in *ompR* were linked with changes in the *E. coli* outer-membrane-protein profile and with the notion that OmpR regulates iron acquisition [98] and induces the expression of the IutA protein, both critical for UPEC survival in the urinary tract [99,100].

The TCS KguS/KguR is another signaling pathway that is present in UPEC strains and absent in commensal and diarrheagenic *E. coli*, suggesting its contribution to UTI [101]. The role of this two-component regulatory system has been first investigated in vivo, by analyzing the ability of Δ*kguS*/*kguR* double-deleted strains to colonize the mouse urinary tract. Indeed, the absence of this TCS negatively impacts on colonization of the bladder and kidneys. The analysis of genes targeted by the KguS/KguR system was performed by growing bacteria in human urine to simulate the urinary tract environment. The differentially expressed proteins were grouped into three main categories on the basis of their functions: metabolism, cell envelope constituents, and translational machinery. A xylose isomerase and several outer membrane proteins were found to be overexpressed in a Δ*kguR* mutant strain, revealing the pleiotropic functions of this TCS. Furthermore, under different growth conditions, constitutive expression of *kguR* induces the over-expression of the *c5035* gene that belongs to a genomic island (*c5032-5039*) strongly associated with the majority of UPEC strains [101]. Since that UPEC in vivo fitness is impaired when mice are infected with Δ*c5032-5039* bacteria, researchers have hypothesized that the described TCS may favor UPEC virulence through the regulation of *c5032–5039* genes. Indeed, activation of the KguS/KguR regulatory system occurs under anaerobic conditions and is induced by α-ketoglutarate (α-KG) that is converted by a dehydrogenase to succinyl-CoA, which is further transformed to succinate by succinyl-CoA synthetase. In *E. coli*, α-KG is the only five-carbon (C5) dicarboxylate used as an intermediate in the citric acid/tricarboxylic acid (TCA) cycle, a fundamental process for the supply of metabolic intermediates and energy in catabolic and anabolic reactions [101]. Therefore, sensing external α-KG might represent an outstanding and unique strategy for UPEC to activate pathogenic mechanisms in anaerobic niches, given the absence of a functional α-KG dehydrogenase in all *E. coli*. Indeed, in the kidney, proximal tubule cells produce greater quantities of α-KG compared to any other cell type [102], suggesting a role of the KguS/KguR TCS in increasing UPEC fitness during in vivo infection. Moreover, deletion of the histidine kinase determines the downregulation of several genes belonging to the *c5032-5039* genomic island in the presence of α-KG, underlying the importance of phosphorylation of the response regulator KguR.

As mentioned above, a major property of UPEC is to adhere to and invade urothelial cells where it can replicate inside the cytoplasm, forming highly proliferative intracellular communities that rapidly detach and infect neighboring epithelial cells [46,103]. In order to reduce bacterial colonization, the host accelerates exfoliation of the superficial layer of the urothelium, which however carries a risk of promoting bacterial dissemination. This may lead to the onset of chronic cystitis or the establishment of intracellular bacterial reservoirs [83,86,104].

Almost half of pathogenic *E. coli* strains isolated by patients with UTI expresses an α-hemolysin (HlyA), mainly involved in in vivo urothelial damage [105,106]. Moreover, HlyA lyses several mammalian cells after its assembly into a water-filled channel that perforates the cell membrane [26,107,108]. It also negatively modifies the cell–cell and cell–matrix interactions promoting exfoliation [109]. Notably, HlyA expression is directly down-regulated by a TCS named CpxRA [110,111,112], that is critical for enhancing UPEC infection in the urinary tract. The CpxRA TCS is composed of an inner membrane sensor with a histidine kinase activity, named CpxA, and of CpxR, the cytoplasmic response regulator. When the bacterial envelope is subjected to stress, CpxA autophosphorylates and, in turn, phosphorylates CpxR which can regulate gene expression. In addition to HlyA downregulation, CpxR is involved in the control protein folding, reduction of envelope stress [113,114], regulation of biofilm assembly [115,116] promotion of bacterial adherence [76] and motility [110,117], and activation of secretion systems [118]. The CpxRA TCS is known to induce the expression of CpxP, a small periplasmic protein that can inactivate the sensor kinase CpxA [119,120]. In addition, CpxP also interacts with unfolded proteins that are subsequently degraded by the protease DegP in order to control incorrect activation of CpxA in the absence of envelope stress [121].

In a mouse model of transurethral infection, viable Δ*cpxRA* bacteria were reduced in number compared with the WT UTI189 strain [122]. In contrast, absence of CpxP had no effect on in vivo infection. Furthermore, in vitro exposure to molecules inducing generalized stress, such as reactive nitrogen and oxygen radicals or factors damaging the bacterial membrane, did not affect the growth of Δ*cpxRA* and Δ*cpxP* bacteria, underlying how the environmental stress sensed by CpxA is specifically associated with in vivo infection. Nagamatsu K. et al. [123] have found that the human cystitis isolate UTI189 deleted for the response regulator CpxR (Δ*cpxR*) is more hemolytic than its wild-type parental strain due to increased HlyA expression and that the recombinant CpxR protein interacts with the *hlyA* promoter in an electrophoretic mobility shift assay (EMSA). By investigating in vivo bladder colonization by UPEC, it was possible to evidence a direct correlation between the CpxRA function, HlyA production, and exfoliation of the urothelium. The bladder colonization rate at 16 h post infection was significantly reduced using the Δ*cpxR* strain or a strain overexpressing *hylA* (WT/p*hlyCABD*), compared to WT, Δ*hlyA*, Δ*cpxR*Δ*hlyA*, and complemented Δ*cpxR* strains [123]. This indicated that excessive production of the HlyA cytotoxin in the absence of a functional CpxRA TCS negatively impacts the ability of UPEC to colonize the bladder. To measure exfoliation in infected mouse bladders, an elegant approach is to observe the distribution and abundance of binucleated superficial facet cells by DAPI-staining of bladder preparations. Since superficial cells also express high amounts expression of uroplakin III, a marker of terminal differentiation, facet cell abundance can be estimated by uroplakin III quantification in homogenized mouse bladders [123]. By either method, marked reductions in facet urothelial cells were observed in bladders infected with Δ*cpxR* and WT/p*hlyCABD* bacteria. Cellular exfoliation is a mechanism that, on the one hand, eliminates superficial infected urothelial cells and, on the other hand, exposes underlying layers of the urothelium to UPEC with a potentially increased risk of deeper bacterial invasion. For these reasons, it is important to understand the mechanism leading to death and exfoliation of urothelial cells and their significance in terms of host defenses. Exfoliation is governed by different pathways that often involves activation of the caspase family of cysteine proteases, as evidenced in models using *Staphylococcus aureus* infection [124]. In the paper by Nagamatsu et al. [123], it was found that HylA over-expression in Δ*cpxR* bacteria triggers, in human cultured urothelial cells, a proinflammatory form of cell death called pyroptosis, through activation of Caspase-1/Caspase-4 and release of IL-1α and IL-1β, as evidenced using chemical inhibitors or siRNA-mediated knockdown of Caspase-1, Caspase-4, and NLRP3. Indeed, these treatments determined a significant reduction in the release of cytoplasmatic lactate dehydrogenase (LDH), IL-1α, and IL-1β from urothelial cells infected with UPEC Δ*cpxR* and WT/p*hlyCABD* strains [123]. Furthermore, fluorescence microscopy revealed the activation of Caspase-1/Caspase-4 as HylA-overexpressing bacteria were surrounded by visible speckles which, conversely, were not visible in Δ*hlyA* or Δ*cpxRΔhlyA*-infected cells. These results were also confirmed in the in vivo UTI model by increased expression of *mCasp11*, *Nlrp3*, *Il1α*, and *Il1β* genes and cleaved Caspase-1 in bladders of mice infected with Δ*cpxR* bacteria [123]. Administration of a Caspase-1/mCaspase-11 inhibitor to mice infected with the Δ*cpxR* strain restored acute virulence of this mutant to WT levels, concomitantly with a reduction of epithelial exfoliation and inflammatory infiltrates [123]. Collectively, these data show that downregulation of *hylA* expression by the CpxRA TCS is fundamental for UPEC to evade host defenses associated with pyroptotic cell death, increased exfoliation, and induction of inflammatory responses.

The *E. coli* BarA-UvrY TCS has been previously described as a signaling mechanism used to withstand osmotic stress, as shown using *barA* mutants [125,126]. In addition, the BarA-UvrY system regulates expression of the *csrA* gene, whose product controls flagellar assembly, carbon metabolism, and biofilm formation [125,126,127,128,129]. In mouse models of ascending UTI, it has been shown that bacteria devoid of *barA* are impaired in their ability to colonize the bladder and the kidneys [130]. Similarly, *uvrY*-deleted UPEC were impaired in their ability to colonize the mouse bladder and invade urothelial cells in vitro [130]. Furthermore, when this TCS is active, cytotoxicity for human kidney cells is augmented, as exemplified by observations that supernatants from the *uvrY*-deleted strain are unable to affect cell viability. Conversely, the effects of *barA* mutation are not as evident as those described for *uvrY* in this system [130].

### 2.2. Pseudomonas aeruginosa

*Pseudomonas aeruginosa* is an opportunistic pathogen that is the third most common cause of UTI. These infections can be acute or chronic and is often difficult to eradicate because of antibiotic resistance. Acute infections are often characterized by a rapid progression [131], while chronic infections appear to be associated with slower bacterial growth, biofilm formation, and antibiotic resistance [132]. Furthermore, this microorganism is linked to infections in immunocompromised patients [133,134]. A multitude of virulence factors, particularly those associated with bacterial colonization and tissue invasion, play important roles in the pathogenesis of *P. aeruginosa* UTI. The main *P. aeruginosa* extracellular appendages are fimbriae and pili that are required for the initial adhesion to host surfaces [135,136], while polysaccharides are involved in maintenance of bacterial communities, including biofilm formation [136,137]. The genome of *P. aeruginosa* contains numerous TCSs which allow bacteria to recognize and respond to environmental signals in a highly complex manner. The best studied TCSs in the context of *P. aeruginosa* pathogenesis are associated with biofilm formation, motility, and production of cytotoxins such as pyocyanin [138].

Biofilms are complex communities of bacterial cells enclosed in an extracellular matrix composed of lipids, polysaccharides, and extracellular DNA (eDNA) [139]. Biofilm assembly is the result of important stages involving reversible attachment, irreversible adherence to surfaces, microcolony formation, maturation, and dispersion. *P. aeruginosa* produces robust biofilms on the surface of human tissues and implanted devices and extracellular polysaccharides, such as Pel, Psl, and alginate, are of major importance in the formation and stability of a microbial biofilm community. Biofilm formation enables the pathogen to become tightly associated with a favorable habitat and to further evade the host immune attack [140]. In UTI, such as CAUTI, biofilms formed by *P. aeruginosa* seem not to require alginate. A model has been proposed whereby pyocyanin, a secreted virulence factor, may promote biofilm formation by increasing the viscosity of eDNA [141].

In this context, TCSs control the change from a planktonic to a sessile lifestyle and vice versa in response to external stimuli by differently regulating the expression of several virulence genes. The initial attachment of bacteria to biotic or abiotic surfaces is an important step in biofilm assembly, in which several surface-exposed components, such as flagella, type IV pili and fimbriae, are involved (Table 4). The TCS PilSR regulates, for example, type IV pilus expression [142], while the FleSR TCS controls the formation of flagella, which increase the hydrophobicity of the bacterial surface and facilitate reversible binding to abiotic surfaces [143]. An additional TCS involved in the initial phases of *P. aeruginosa* biofilm formation is the Roc system, where RocS1 and RocA1 are sensor HK and RR, respectively. The Roc system favors the expression of *cupB* and *cupC* gene clusters, each containing a minimal set of genes required for the expression of adhesive fimbrial organelles belonging to the chaperone/usher family [144]. The BfiSR TCS is, instead, involved in the bacterial irreversible attachment through transcriptional regulation of the RNase G, which modulates the levels of a small RNA (*rsmZ*), belonging to a well-characterized small RNA-based regulatory system [145]. The KinB-AlgB TCS (in which KinB is the HK and AlgB the transcriptional activator) is involved in the direct regulation of *algD* [146], a gene required for alginate biosynthesis and in the onset of mucoid phenotypes as suggested by acute pneumonia murine models [147]. Moreover, an AlgB-independent role of KinB has been demonstrated in models of acute infections in zebrafish embryos [148]. BfmR is a response regulator that modulates the release of eDNA, which is required for biofilm maturation and integrity [149] and the deletion of its cognate sensor regulator BfmS causes a significant reduction of the recovered bacteria from infected murine lungs [150]. The high rate of BfmR phosphorylation and enhanced chronic infection state has been further linked to single point mutations found in the sensor BfmS of clinical isolates [151]. PprAB, another important TCS, controls the expression of the *cupE* gene cluster responsible for the regulation of cell-to-cell connections not only during microcolony formation but also in the final maturation of biofilms [152]. Finally, modulation of rhamnolipids and other signaling molecules is associated with the ability of the TCS BqsSR to promote biofilm dispersal, as assessed by experimental approaches performed with Δ*bqsS* and Δ*bqsR* bacteria [153]. In the context of UTI, rhamnolipid expression is upregulated under the iron-limiting conditions of the urinary tract, enabling *P. aeruginosa* ascension and persistence [154].

*P. aeruginosa* uses three different types of motility to colonize the host: swimming, swarming, and twitching. The role of *P. aeruginosa* motility in UTI is not clear. A report dealing with a limited number of isolates showed an increased bacterial ability to swarm in UTI compared to CAUTI, whereas in the context of CAUTI, swarming was lower in chronic conditions compared to acute ones [155]. The swimming process involves the rotation of a single flagellum, while swarming requires intercellular coordination among bacteria to progress on surfaces, and twitching involves continuous length changes of type IV pili. Several TCSs have been also described referring to motility (Table 4). The FleSR TCS governs flagellar biosynthesis while the GacSA system works together with other membrane sensors to finely regulate sRNAs associated with intracellular signals triggering in acute infection [143,156]. Other studies demonstrated how the CarSR regulatory system regulates swarming motility by sensing external Ca^2+^ concentrations [157] and how PilSR is responsible of pilus- and flagellum-dependent swimming motility [142]. In contrast to flagella, type IV pili are used by *P. aeruginosa* to move by alternating ATP-dependent elongation and retraction phases [158]. The FimS-AlgR, for example, controls the twitching motility by enhancing the expression of genes involved in pili assembly [159], whereas phosphorylation of the response regulator PilG is useful to facilitate pilus extension [160].

*P. aeruginosa* produces pyocyanin whose cytotoxic effects have been well characterized [161]. Pyocyanin production is regulated by several Quorum Sensing (QS) systems, but the role played by TCSs is also crucial (Table 4). The association of GacSA with the sensors LadS and RetS is important in the expression of *phz* operons encoding for the pyocyanin precursors such as chorismic acid [162]. The BqsSR system positively regulates the 2-heptyl-3-hydroxy-4-quinolone (PQS) that is essential for pyocyanin production [153], whereas a non-functional BqsSR system decreases pyocyanin synthesis [153]. The AlgZR TCS regulates indirectly pyocyanin production through the repressor CzcR and an interesting role has been ascribed to phosphorylation of the regulator AlgZR in acute murine wound and pneumonia models of infections [163].

In the course of pathogenesis, *P. aeruginosa* secretes numerous toxins and hydrolytic enzymes through different types of secretion systems. Secretion system type II and III are the most important. The type II system favors the secretion of the chitin-binding protein E and D (CbpE and CbpD, respectively), elastase or exotoxin A (also named as ToxA) [164,165]. Conversely, the type III system promotes the secretion of ExoS, ExoT, ExoU, and ExoU by playing, in this manner, a relevant role in host damage and immune response dysfunction. Clinical *P. aeruginosa* isolates from UTI have revealed higher levels of ExoS compared to tracheal isolates [166]. The GtrS-GltR TCS is engaged in ToxA production [167] and *gtrS* mutant bacteria are unable to greatly infect murine lungs [168], while the GacSA system controls elastase and type III secretion systems [169]. Moreover, the Roc system is involved in the production of ExoY and ExoT toxins [169], compared to CbrAB that determines the synthesis also of ExoS toxin [170]. Furthermore, the role of the sensor kinase CbrA has been investigated in murine peritoneal and lung infection models by showing a loss of virulence for *cbrA* mutant and ruling out the involvement of alterations in bacterial motility, biofilm formation, and cytotoxicity [171]. Contrarily, the FimS-AlgR system negatively regulates type III secretion [172] (Table 4).

Antimicrobial resistance is not only associated with both biofilm formation, but also intracellular persistence [173]. It has been demonstrated that *P. aeruginosa* can survive inside human bladder epithelial cells and stably reside in intracellular reservoirs by means of the two-component regulator AlgZR [174]. As mentioned above, AlgR is an important virulence factor regulator, controlling type IV pilus-dependent twitching motility and alginate production (via regulation of *algC* and *algD* promoters). It also negatively regulates biofilm formation, quorum sensing, and anaerobic metabolism [175]. Both laboratory and clinical strains were found to remain alive intracellularly for up to 48 h, as shown by gentamicin protection assays and by confocal imaging revealing *Pseudomonas aeruginosa* co-localization with the intracellular marker of endocytosis LAMP-1 [174]. Deletion of the *algR* gene is associated with decreased intracellular survival, compared to WT bacteria. Like other two-component response regulators [53], AlgR is activated after being phosphorylated by the histidine kinase AlgZ (or FimS) [175]. Deletion of *algZ* results in reduced invasion and intracellular survival, suggesting that phosphorylation of AlgR might be involved in these phenomena. An interesting mutational approach to investigate the activation of a response regulator by its cognate histidine kinase is to create a single point mutation in the predicted RR phosphorylation site. Mutants with a non-phosphorylated (D54N) form of AlgR (AlgR phosphoablative, locked into the constitutive, non-activated state) were defective in bladder cell invasion, while a phosphomimetic (D54E) mutant locked into the activated state invaded cells similarly as WT bacteria [174].

Bacterial adaptation to the intracellular host environment is associated with transcriptional changes that can be studied in depth by using transcriptomic, or RNA-seq, analysis [176], although it is not always technically easy to recover sufficient amounts of bacterial RNA for this kind of studies. After invasion of bladder epithelial cells, wild-type bacteria upregulate genes encoding virulence factors, such as the type III secretion system and iron acquisition proteins such as pyocyanin and pyoverdin [177,178], while downregulate genes related to arginine catabolism and lipopolysaccharide synthesis, which were previously found to be overexpressed by extracellular bacteria in a murine pneumonia model [178]. Conversely, by comparing transcriptional profiles of WT and Δ*algR* bacteria, seven genes involved in protein translation and in transmembrane protein synthesis were shown to be downregulated in the Δ*algR* strain. Moreover, low amounts of ribosomal RNA (rRNA) were detected in the Δ*algR* strain, suggesting that even in the first stages of *P. aeruginosa* internalization into bladder cells, bacteria without the response regulator AlgR may be incapable of surviving in the intracellular compartments because of deficiencies in their translational machinery [174].

On the other hand, in the course of bacterial infection, the host changes its transcriptional activity to counteract the invading pathogen. In light of this, information on the host, in addition to the pathogen, transcriptional responses may be useful to understand the mechanisms of intracellular infection by *P. aeruginosa*. Dual RNA-seq studies indicated that activation of NF-κB-dependent genes in bladder epithelial cells is exploited by *P. aeruginosa* to survive intracellularly [174]. For example, blockade of NF-κB signaling with chemical inhibitors reduced intracellular survival of wild-type *P. aeruginosa*. NF-κB-dependent bacterial survival in bladder cells is paradoxical, since the same NF-κB pathway was previously found to be required for inflammatory responses and clearance of extracellular *P. aeruginosa* in a pneumonia model [178].

To study the role of the AlgZR TCS in vivo, mice were transurethrally infected with *P. aeruginosa* and bladders were harvested to measure colony forming units (CFU). After 2 h of infection, a high number of WT bacteria was recovered intracellularly and was found to persist for up to 7 days in almost all infected mice. On the contrary, deletion of *algR* resulted in low and transient intracellular bacterial burden in the bladder [174].

In light of these data, the authors went on to demonstrate that *P. aeruginosa* survival inside epithelial cells contributes to the onset and recurrence of chronic infection. Furthermore, persistent infections by uropathogens are strictly related to antibiotic resistance [179] and, in the case of *P. aeruginosa*, the discovery of therapies targeting the host inflammatory pathways such as NF-κB signaling could be an intriguing approach to control infections of the urinary tract.

### 2.3. Enterococcus faecalis

*Enterococcus faecalis* is a Gram-positive bacterium responsible for CAUTI. Implantation of a urinary catheter in the bladder evokes histological changes [180], inflammation [181], and the release of fibrinogen which coats the catheter [182]. Binding to the extracellular matrix has been described for different enterococcal proteins, such as Ace (adhesin of collagen from enterococci) and *E. faecalis* surface proteins (Fss). While Ace binds to collagen types I and IV, laminin, and dentin [183,184], Fss proteins bind to fibrinogen [185]. Recent studies demonstrated that enterococcal membrane glycolipids recognize the glycosaminoglycans heparin and heparan sulfate present on human colonic cells to mediate bacterial adhesion to host tissues [186]. *E. faecalis* harbors two pilin gene clusters (PGCs), named *ebp* locus (standing for endocarditis and biofilm-associated pili) [187] and the *bee* locus (biofilm enhancer in enterococci) [188], both contributing to the pathogenesis of endocarditis and urinary tract infection. Esp proteins facilitate the *E. faecalis* adherence to fibrinogen and collagen receptors present in bladder cells in the mouse model. In addition, in vivo studies show that Ebp regulate the biofilm formation during CAUTI [37]. Gelatinase is, instead, a protease used by *E. faecalis* to control biofilm formation and its expression is increased by the fsr TCS which is composed of four genes (*fsrA*, *fsrB*, *fsrD*, and *fsrC*) [189]. This system is activated in response to a peptide lactone, which is encoded by *fsrD* [189]. As far as we know, the two-component regulatory system GrvRS, which directly controls *ace* transcription [190], is the only one to have been investigated in UTI. Bacteria deleted for *grvR* (Δ*grvR*) are impaired in their ability to colonize the urinary tract and, conversely, complementation of Δ*grvR* restores in vivo virulence. Moreover, the absence of GrvR determines a reduction in binding to Ace extracellular matrix targets and biofilm formation.

### 2.4. Streptococcus agalactiae

Group B streptococcus (or GBS) is a Gram-positive commensal of the human gastrointestinal and genital tract flora in almost 30% of healthy women [191,192]. This bacterium is one of the leading causes of infection in newborns [193] and adults with chronic pathologies [194]. It can cause sepsis and meningitis in neonates after infection in utero or during passage through the birth canal in the first week of life (early onset disease). Neonates can also acquire infection at later times and develop late-onset disease, predominantly associated with meningitis. In addition to neonatal infection and sepsis in adults, this pathogen can be also responsible for UTI, accounting for approximately 2–3% of all UTI [5,195]. GBS UTI includes asymptomatic bacteriuria (ABU), cystitis, pyelonephritis, and urosepsis [196,197]. Furthermore, GBS UTI during pregnancy carries a high risk of chorioamnionitis and premature labor [198,199]. While the actual prevalence of GBS UTI is uncertain, GBS ABU detected during pregnancy is a clear indication of maternal genital tract colonization [199,200]. Indeed, GBS ABU has been associated with a high risk for early-onset disease (EOD) in newborns [199,201] and, for this reason, must be taken into consideration for intrapartum antibiotic chemoprophylaxis in conjunction with other factors [202]. In contrast to other pathological conditions caused by GBS, the clinical and molecular mechanisms of GBS UTI are not well understood. Some studies have shown that the secretion of inflammatory mediators, in mouse models of transurethral infection is induced during serotype III GBS strains infection [195,203,204], but only few studies have described the role played by known GBS-virulence factors in this context. For example, sialic-acid residues of the capsular polysaccharide are likely to play a role for infection of the urinary tract [202,205], whereas β-hemolysin/cytolysin (β-h/c) can elicit a strong neutrophil response in the murine bladder because of its cytotoxic effect on uroepithelial cells [16].

As other organisms, GBS encounters different environmental niches and uses several systems to sense external signals in order to ensure its survival and efficiently colonize the host [206,207]. GBS has more than 20 different two-component systems that have been classified in different categories on the basis of their functions, such as resistance to antibiotics, adhesion to the mucosal surfaces, metabolic regulation, and pathogenesis [208]. The majority of widely adopted TCS by GBS has been investigated in mouse infection models (Table 5).

One of the most studied TCS in GBS is the CovRS regulatory system where the sensor histidine kinase CovS senses external signals and activates the response regulator CovR [220]. This system directly or indirectly regulates the expression of different genes [221], including those encoding virulence factors, such as β-hemolysin/cytolysin (β-h/c) [222], HvgA [223], PbsP [209,224,225,226], and other ones. Furthermore, *pbsP* is subjected to the transcriptional regulation mediated by the recently discovered SaeRS TCS that greatly enhances its expression by rendering GBS more virulent in vivo [216]. Studies on SaeRS correlation with *pbsP* are currently underway among different clonotypes and in other in vivo infection models, given the importance of this adhesin in GBS pathogenesis [209,224,225,226].

CovR is particularly involved in favoring the transition from commensalism to virulence by controlling the expression of more than 150 genes. Until now, only a paper briefly describes the in vivo contribution of this TCS to the onset of UTI [227]. Infection assays using a *covR*-deficient mutant demonstrate significant attenuation in the ability of GBS to colonize the bladder and urine in a mouse model. Furthermore, the levels of pro-inflammatory cytokines, such as interleukin 6, 17A, 12, and chemokines, are lower in response to bacteria lacking CovR compared with the WT strain upon bladder infection. In contrast, other innate immune mediators are equally overexpressed by both WT and *covR*-deficient strains. In vitro experiments on bladder cells corroborate the idea that CovR is involved in cellular adhesion and invasion and in acute GBS-induced cytotoxicity. Indeed, fluorescence images revealed co-localization of Δ*covR* bacteria and Caspase 3 (an executioner of apoptosis), a feature that correlates with the hyper-hemolytic phenotype of the mutant strain. This observation is in line with the described role of β-h/c in *S. agalactiae* UTI [16]. As a pathogenic strategy, GBS is able to alter the expression of CovR in vivo to finely modulate virulence genes expression on the basis of the host niches encountered [228]. This CovR properties are in line with other in vivo models of GBS infection. For instance, in murine sepsis models, the absence of CovR results in the recovery of increased numbers of bacteria in infected brains [210]. Moreover, *covR*-deficient bacteria are less invasive in human vaginal epithelial cells [211].

### 2.5. Proteus Mirabilis

*Proteus mirabilis* is a leading cause of pyelonephritis, urolithiasis and is, particularly, associated with long-term catheterization [229,230]. *Proteus* has several virulence factors such as fimbriae or appendages mediating adherence to cells of the urinary tract and also to catheters [231,232,233]. Furthermore, this pathogen produces urease that uses ammonia as substrate by increasing pH with the subsequent precipitation of calcium and magnesium ions and the formation of urinary stones that can reduce urinary flow with consequent tissue damage [234,235]. The precipitation of minerals may further promote bacterial adherence to the catheter, laying the foundations for biofilm formation [36,236]. Subsequently, bacteria embedded in urinary stones may evade the host immune response and/or the efficacy of antibiotics [237]. These conclusions were reached using a mouse model of UTI where the absence of the gene encoding for urease significantly decreased the number of recovered bacteria from kidney and bladder [238]. *P. mirabilis* migrates along catheters by means of a swarming process to migrate along the urinary tract [239,240], even though non-swarming mutants progress only on latex catheters coated with hydrogels [241]. Moreover, in *P. mirabilis* biofilms, swarm cells have been observed and proposed as mediators of bacterial dispersal from catheter to the urinary tract [242]. As far as we know, few TCSs have been characterized in *P. mirabilis*, but not in the context of murine urinary tract models of infection. The RppAB TCS regulates the synthesis of LPS and flagella. RppA is, indeed, a regulator of *arnA* gene that encodes for a bacterial decarboxylase useful for LPS modification [243]. A mutated *rppA* gene is also linked to a more evident swarming phenotype [244]. Another recent paper has also highlighted the role played by the QseEF TCS in modulating swarming motility. In general, the activated sensor kinase QseE determines the phosphorylation of the regulator QseF that, in turn, activates the expression of GlmY together with CheA and RcsB, two antagonist regulators of flagellum production [245]. A recent case report has described evidence of acquisition of resistance to imipenem and amikacin in a *Proteus mirabilis* clinical isolate, not previously showing this feature. Sequencing revealed the appearance of a point mutation (His-208-Pro) in the HAMP domain of the CpxA sensor [246]. This observation is in line with that seen in the CpxA of other microorganisms revealing resistance to β-lactams and major expression of efflux pump [247] by indicating how relevant is the role played by TCS in antibiotic resistance [248].

## 3. Conclusions

UTI represents a widespread health emergency [1,8]. Uropathogens produce a wide range of virulence factors that are essential for early attachment to host tissues and for the establishment of infection [17]. Furthermore, the majority of UTI is largely associated with antibiotic resistance [19]. Treatment of patients with antimicrobial drugs is progressively leading to the alteration of the normal microbiota leaving open field to recurrent UTI [19,20]. Intense efforts are, therefore, needed to identify selective targets that may be blocked in order to dysregulate bacterial pathogenetic mechanisms. A special feature of bacterial pathogenesis is the continuous change of transcriptomic profiles by means of TCS to enhance survival, proliferation, and spreading in the host. These systems combine signal recognition, signal transduction, and gene activation by using a sensor and a response regulator [53,249]. More than one TCS is likely involved in the onset of infection and extensive networking among them is required to produce complex responses that allow bacteria to endure in hostile niches. Notably, two-component proteins from different bacterial species share similar amino acid motifs located near the activation site [249] and a high degree of structural homology [250], leading us to hypothesize that deeper knowledge of these general features may be extremely relevant for the development of new therapeutic strategies [251]. Main molecular targets may include auto-phosphorylation sites in the sensor, phosphoryl transfer to the response regulator, and binding of RR to the gene promoter [251]. For this reason, it is of considerable importance to obtain a more detailed characterization of TCS signaling pathways under both in vitro and in vivo conditions with the final goal of controlling UTI.

## Figures and Tables

**Table 1:** List of TCS involved in in vivo models of UTI and discussed in this review.

TCS	Pathogen
AlgZR	*P. aeruginosa*
EnvZ-OmpR	UPEC
KguSR	UPEC
CpxRA	UPEC
BarA-UvrY	UPEC
GrvRS	*E. faecalis*
CovRS	GBS

**Table 2:** Virulence factors of Uropathogenic *E. coli* (UPEC) in UTI.

Surface Virulence Factors	Main Pathogenic Functions
Type 1 fimbriae	Enhanced bacterial survival; Stimulation of inflammation; Promotion of invasion; Biofilm growth; Binding to uroplakin
P fimbriae	Binding to kidney glycosphingolipids;
	Bacterial colonization of tubular epithelium
S fimbriae	Binding to the lower human urinary tract and kidney; Bacterial dissemination
Fimbrial Dr	Binding to type IV collagen and to decay-accelerating factor (DAF)
Adhesin Afa	Establishment of chronic and/or recurrent infection
Flagella	Bacterial motility; Bacterial ascension from the bladder to kidneys
**Secreted virulence factors**	**Main pathogenic functions**
α-haemolysin (HlyA)	Lysis of erythrocytes and nucleated cells; Induction of apoptosis of host cells; Exfoliation of bladder epithelial cells
Cytotoxic necrotizing factor 1 (CNF1)	Kidney invasion; Interference with phagocytosis; Exfoliation of bladder cells
Secreted autotransporter toxin (SAT)	Damage of bladder or kidney cells

**Table 3:** Functional properties of TCSs reported in UPEC section of this review.

TCS	Functional Characterization
EnvZ-OmpR	Sustenance of bacterial growth in urine; Promotion of UPEC ascension along the urinary tract
KguSR	Colonization of bladder; Regulation of metabolism and cell envelope constituents; Regulation of the *c5032-5039* genomic island;
	Sensing of α-ketoglutarate in the urinary tract
CpxRA	Resistance to environmental stress; Regulation of HylA expression; Induction of urothelial exfoliation
BarA-UvrY	Colonization of the bladder and the kidneys; Invasion of urothelial cells; Induction of cellular cytotoxicity

**Table 4:** Set of the main TCSs associated with virulence in *P. aeruginosa*.

TCS	Step in Pathogenesis	Virulence Factor
PilSR FleSR Roc system BfiSR KinB-AlgB BfmSR PprAB BqsSR	Biofilm formation	Type IV pili Flagella Fimbriae RnaseG Alginate eDNA Fimbriae Rhamnolipids
FleSR GacSA CarsR PilSR FimS-AlgR	Motility	Flagella sRNAs CarP Pili Pili
GacSA-LadS-RetS BqsSR AlgZR	Pigment formation	Pyocyanin
		Pyocyanin
		Pyocyanin
GtrS-GltR Roc system CbrAB GacSA	Release of extracellular virulence factors	ToxA ExoY and Exo T ExoS
		Elastase

**Table 5:** Two-component systems (TCSs) of group B streptococcus (GBS) studied in in vivo mouse models different from UTI.

TCS	In Vivo Mouse Model	References
CovRS	Meningitis	[209]
CovRS	Meningitis	[210]
CovRS	Vaginal	[211]
HssRS	Systemic infection	[212]
LtdRS	Vaginal	[213]
RgfAC	Meningitis	[214,215]
SaeRS	Vaginal	[216]
FspSR	Vaginal	[214]
LiaSR	Sepsis/pneumonia	[217]
NsrRK/BceRS	Intraperitoneal	[218]
CiaRH	Meningitis	[219]

## Data Availability

Not applicable.
